# Antibodies That Induce Phagocytosis of Malaria Infected Erythrocytes: Effect of HIV Infection and Correlation with Clinical Outcomes

**DOI:** 10.1371/journal.pone.0022491

**Published:** 2011-07-19

**Authors:** Ricardo Ataíde, Victor Mwapasa, Malcolm E. Molyneux, Steven R. Meshnick, Stephen J. Rogerson

**Affiliations:** 1 Department of Medicine (RMH/WH), University of Melbourne, Post Office Royal Melbourne Hospital, Melbourne, Victoria, Australia; 2 Graduate Program in Areas of Basic and Applied Biology (GABBA), Universidade do Porto, Porto, Portugal; 3 Malawi-Liverpool-Wellcome Trust Clinical Research Programme, College of Medicine, University of Malawi, Blantyre, Malawi; 4 Department of Community Health, College of Medicine, University of Malawi, Blantyre, Malawi; 5 School of Tropical Medicine, University of Liverpool, Liverpool, United Kingdom; 6 Department of Microbiology and Immunology, University of North Carolina, Chapel Hill, North Carolina, United States of America; Universidade Federal de Minas Gerais, Brazil

## Abstract

HIV infection increases the burden of disease of malaria in pregnancy, in part by impairing the development of immunity. We measured total IgG and phagocytic antibodies against variant surface antigens of placental-type CS2 parasites in 187 secundigravidae (65% HIV infected). In women with placental malaria infection, phagocytic antibodies to CS2_VSA_ were decreased in the presence of HIV (*p* = 0.011) and correlated positively with infant birth weight (coef = 3.57, *p* = 0.025), whereas total IgG to CS2_VSA_ did not. Phagocytic antibodies to CS2_VSA_ are valuable tools to study acquired immunity to malaria in the context of HIV co-infection. Secundigravidae may be an informative group for identification of correlates of immunity.

## Introduction

Malaria in pregnancy is a major cause of mortality and morbidity in malaria endemic regions, and the concomitant presence of HIV worsens the adverse clinical outcomes with which it is associated [1] When antibodies towards the variant surface antigens of the placental-type parasite line CS2 (CS2_VSA_) were analysed in a cohort of primigravidae from Malawi it became apparent that HIV had a bigger impact on antibodies that contribute to phagocytosis (hereafter mentioned as phagocytic antibodies), particularly the acquisition phase of those antibodies, than on the total IgG to CS2_VSA_ [2] (also suggested by [3]). In these primigravidae, unlike multigravidae (reviewed in [4]) antibody levels were not associated with infant birth weight or maternal haemoglobin concentration. This lack of association was attributed to the importance of the timing of the antibody acquisition rather than to the levels of antibody achieved, and subsequently measured, at the end of the pregnancy. It was speculated, also, that in subsequent pregnancies the difference between the effect of HIV on phagocytic antibodies and on total IgG to CS2_VSA_ would become more apparent, since HIV was shown to affect the acquisition of phagocytic antibodies more than the recall or maintenance of these responses. With this in mind, we studied the function (opsonisation, measured as induction of phagocytosis) and level (total IgG) of anti-CS2_VSA_ antibodies in secundigravidae (women in their second pregnancy) from the same cohort in which we had previously studied the relationship between placental malaria and HIV in primigravid cohort members. We found that in secundigravidae, concentrations of circulating phagocytic antibodies were modulated by the presence of HIV, whereas the total levels of IgG to CS2_VSA_ were not. Among women with placental malaria at the end of their second pregnancy, the measured levels of phagocytic antibodies to CS2_VSA_ at delivery correlated positively with infant birth weight.

## Methods

### Ethics statement

Ethical clearance for the study was provided by the College of Medicine Research Ethics Committee, University of Malawi, the Melbourne Health Human Research Ethics Committee, Melbourne, Australia, and the Institutional Review Board of the University of North Carolina, Chapel Hill, NC, USA.

### Study samples

Serum samples came from a study group which has previously been described [5], [6]. In brief, women in late third trimester of pregnancy consented to studies including HIV testing, and samples of peripheral blood and placenta were collected. Only secundigravid women for whom histology were reported to have been performed (n = 187) were included in the present study. All samples were heat inactivated before use.

Placental tissue, collected at delivery, was fixed in formalin and Giemsa-stained sections were examined histologically as previously described [7]. Placental malaria was considered to be present if there was evidence of *Plasmodium falciparum* parasites or pigmented monocytes or pigment in fibrin deposits in the placenta, irrespective of peripheral blood parasitaemia.

We combined histology findings with results of microscopy of Giemsa-stained placental and peripheral blood thick films to allocate women to one of four groups: *No Malaria* (no evidence of past or current malaria, either in blood films or placental histology), *Acute Infection* (pRBC on placental histology, without malaria pigment deposition), *Chronic Infection* (both pRBC and malaria pigment deposits on histology) and *Past Infection* (placental malaria pigment deposits, without pRBC on histology or on blood films). We excluded from the grouping analysis women who presented only with peripheral parasitaemia. All infections were with *P. falciparum*.

### Cell culture and Parasites

Thp-1 cells, obtained from the ATCC (catalog number: TIB-202™), and the parasite lines CS2 and CS2-GFP were cultured and maintained as previously described [2]. CS2 is similar to placental-type isolates, binding to CSA and being recognised by serum in a pregnancy-specific and gravidity-specific manner; CS2-GFP shares these properties, and has been transfected to express green fluorescent protein, using published techniques [8].

### Antibody measurements: Assays of IgG to CS2_VSA_ and the phagocytosis assay

IgG to CS2_VSA_was measured as described elsewhere [2] with minor modifications. In summary, mid to late trophozoite-stage CS2 parasitised red blood cells (pRBC) at 1–10% parasitaemia were resuspended at 0.1% hematocrit in PBS with 1% newborn calf serum (NCS) and incubated with test serum at 1/20 dilution for 30 minutes in a 96-well plate at room temperature. Rabbit anti-human IgG (Dako) 1/100 in PBS/ NCS and Alexafluor 488-conjugated donkey anti-rabbit IgG (Invitrogen) at 1/500 dilution in PBS/NCS containing 10 µg/ml EtBr were used as secondary and tertiary antibodies, respectively. Cells were analysed on a FACSCalibur flow cytometer with BD CellQuest™ software version 5.2.1 (BD Biosciences). The positive control was a pool of serum with known high antibody recognition to CS2. Negative controls were from unexposed Australian donors. MFI values for RBC alone were subtracted from MFI of pRBC to obtain the CS2_VSA_ specific MFI. This was then converted into a percentage of the positive control MFI, using the formula [9]:
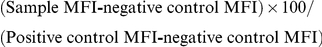
The phagocytosis assay for the assessment of anti-CS2_VSA_ antibodies' function was performed exactly as previously described [2] with the only modification being the measurement of samples in duplicate rather than in triplicate. In short, CS2-GFP pRBC were opsonised with sample or control serum at 1/10 dilution in 96-well plates, washed thrice, added to uThp-1 cells at a 1∶10 ratio and left to phagocytose for 40 min in a humidified incubator with 5% CO_2_ at 37°C. After lysis of free pRBC with FACS Lysing solution, cells were washed thrice, resuspended in 2% Paraformaldehyde in PBS and acquired on a FACSCalibur flow cytometer with BD CellQuest™ software. A minimum of 10,000 cells were acquired.

### Database analysis and statistical analysis

Results were analysed in Stata v9.2 (Stata Corporation, College Station, TX) or GraphPad Prism v 4.2 (GraphPad Software, Inc.). Age, birth weight and maternal haemoglobin levels were normally distributed and Student's t-tests were applied and *p*-values are given. Total IgG to CS2_VSA_ and phagocytic antibodies both given as percentage of positive controls (the positive controls being a pool of sera with known high antibody recognition to CS2) were not normally distributed, and data were analysed using Mann-Whitney rank sum tests. Medians and interquartile ranges (IQR) are given together with the correspondent *p*-value. All other variables were categorical. Multiple linear regression models were used to seek correlations between continuous and categorical variables and regression coefficients and *p*-values are given.

## Results

### Study cohort characteristics according to HIV and Placental malaria status

One hundred and eighty seven samples were available. Forty two percent of participants had placental malaria and 65% were HIV infected (Table 1). Most women (62%) were anaemic, and mean haemoglobin concentration ± standard deviation (SD) was 10.6±2.1 g/dL. Haemoglobin levels were not associated in a univariate analysis with placental malaria infection or with HIV status in this study group (Table 1). Infant birth weight did not vary with placental malaria infection, but was significantly lower among HIV-positive women (P<0.001, Table 1). Women with evidence of placental malaria infection were significantly younger than uninfected women (P = 0.002; Table 1). Women were not more likely to have evidence of placental malaria infection with either the presence or absence of a concomitant HIV infection (logistic regression: odds ratio = 0.79, *p* = 0.341).

**Table 1:** Characteristics of the cohort and associations with HIV and placental malaria.

			Age			Maternal Hb level (g/dL)^1^			Infant Bw (g)^2^	
		n [%]	Mean (SD)	p-value	n [%]	Mean (SD)	p-value	n [%]	Mean (SD)	p-value
**Entire Cohort**		187 [100]	22.5 (3.1)		129 [100]	10.6 (2.14)		181 [100]	2852 (518)	
**HIV**	**negative**	65 [35]	22.1(2.3)		30 [23]	10.7 (2.1)		61 [34]	3091 (452)	
	**positive**	122 [65]	22.7 (3.4)	0.197	99 [77]	10.6 (2.2)	0.934	120 [66]	2731 (510)	*<0.001*
**Malaria infection**	**negative**	89 [48]	23.1 (3.5)		58 [45]	10.9 (2.2)		87 [44]	2852 (561)	
	**positive**	79 [42]	21.6 (2.3)	*0.002*	61 [47]	10.5 (2.0)	0.252	78 [43]	2865 (438)	0.875
	**Not available**	19 [22]			10 [8]			16 [9]		
**HIV and Malaria:**										
**DOUBLE NEGATIVES** 3		27 [14]	22.3 (2.0)		9 [7]	11.1 (2.4)		26 [14]	3112 (501)	
**DOUBLE POSITIVES** 4		47 [25]	21.5 (2.1)	0.119	42 [33]	10.5 (2.0)	0.404	46 [25]	2714 (390)	<0.001

The data represents numbers of patients (percentages of total for each parameter) and means [standard deviations].

1 Maternal haemoglobin levels.

2 Birth weight of babies at delivery.

3 DOUBLE NEGATIVES are women with neither HIV nor malaria.

4 DOUBLE POSITIVES are women with both HIV and malaria infection. Variables are normally distributed, so Student's t-tests were applied.

### Associations between antibody levels and HIV, placental malaria status, maternal haemoglobin and infants' birth weight

The correlation between the levels of total IgG to CS2_VSA_ and the phagocytic function in this cohort of secundigravid women was similar to previous observations (r^2^ = 0.67) The spread of values for total IgG to CS2_VSA_ was larger than that for phagocytic antibodies.

**Figure 1:**
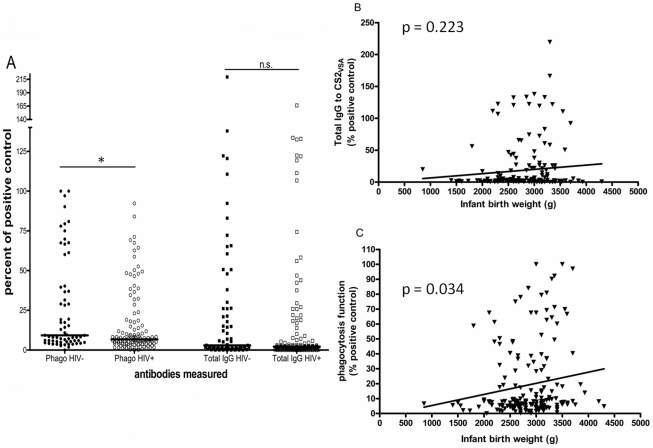
Associations of antibodies measured with HIV and infant birth weight. A) Values for both phagocytic antibodies (circles) and total IgG to CS2_VSA_ (squares) were plotted according to the women's’ HIV status (HIV negative – full symbols; HIV positive – open symbols). Mann-Whitney rank sum tests were performed on HIV positive vs. negative women. *p = 0.010; n.s. ( p = 0.078). Univariate linear regression of total IgG to CS2_VSA_ (B) and phagocytic antibodies (C) and infant birth weight. Both p-values are plotted. Only phagocytic antibodies correlated with infant birth weight (r^2^ = 0.025, p = 0.034).

We compared antibody levels in women with evidence of placental malaria infection (PM+) and without infection (PM−). For phagocytic antibodies levels were higher in PM+ women (median; IQR 11.79; 5.92–79.45) than in PM− women (6.00; 3.81–8.52; *p*<0.001). Similarly, Total IgG to CS2_VSA_ was higher in PM+ women (5.35; 1.77–26.07) than PM− women (1.75; 0.45–3.19; *p*<0.001). Phagocytic antibodies were significantly decreased in HIV infected women (6.72; 4.22–14.78) compared to HIV uninfected women (9.21; 5.36–36.98; *p* = 0.011), whereas total IgG to CS2_VSA_ showed a non-significant difference between HIV-infected women (2.14; 0.74–6.71) and women who were HIV-negative (2.97; 1.29–26.20; *p* = 0.079; Figure 1A). Neither phagocytic antibodies nor total IgG to CS2_VSA_ was correlated with maternal haemoglobin levels. Infant birth weight was also not associated with total IgG to CS2_VSA_ (linear regression (LR): coef = 0.007, *p* = 0.223) (Figure 1B) but it was correlated with phagocytic antibodies (LR: coef = 3.34, *p* = 0.034) (Figure 1C).

A multivariate analysis was performed using HIV status, antibodies and maternal age and weight against infant birth weight and maternal haemoglobin concentration. Women were divided into placental-malaria positive and placental-malaria negative. Total IgG to CS2_VSA_ was not associated with infant birth weight in either group (data not shown). Phagocytic antibodies were not associated with infant birth weight in placental-malaria negative women (LR: coef = 0.42, *p* = 0.902), but remained significantly associated with birth weight, after adjusting for HIV and maternal age and weight, in women with placental malaria (LR: coef = 3.57, *p* = 0.025). Neither antibody measurement was associated with maternal haemoglobin levels in multivariate analyses.

### Antibody parameters and placental malaria status

The levels of total IgG to CS2_VSA_ and of phagocytic antibodies did not vary according to findings on placental histology, (data not shown), being similar in women with acute, chronic and past infection (Anova across groups, Total IgG to CS2_VSA_: *p* = 0.266; phago: *p* = 0.429) Relatively low numbers diminished the power of this analysis to assess the effect of HIV on immunity in different types of placental infection.

**Figure 2:**
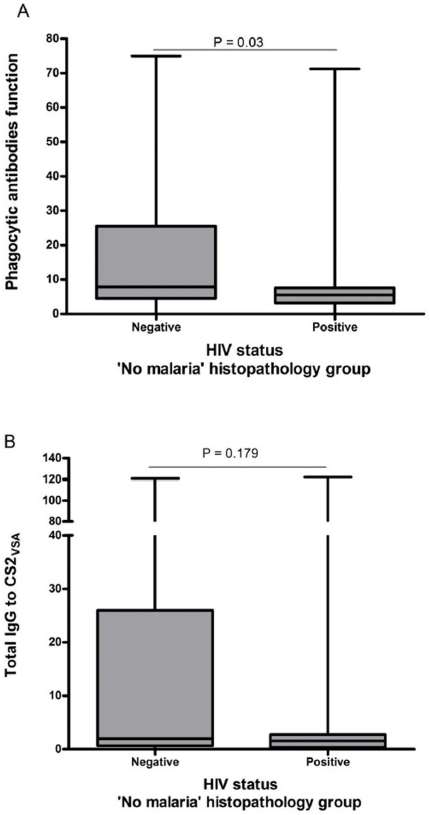
Effect of HIV on the anti-CS2_VSA_ antibody levels in the ‘No Malaria’ histopathology group. In a univariate analysis of women that showed no evidence of either peripheral or placental malaria infection, using Mann-Whitney ranksum tests, phagocytic antibodies (A) are shown to be significantly decreased by HIV infection (z = −2.17, *p* = 0.03). On the contrary total IgG to CS2_VSA_ (B) shows a non-significant decrease in the HIV-positive group (z = −1.35, *p* = 0.179. Box, median and interquartile range; whiskers, lowest and highest values. P values are shown.

In women belonging to the histopathology group ‘No Malaria’ (where no evidence of either peripheral or placental malaria was detected) sufficient numbers were available for an analysis of the effect of HIV (n = 109). HIV significantly affected the function of phagocytic antibodies (z = −2.17, P = 0.03) (Figure 2A), but not the total IgG to CS2_VSA_ (Z = −1.35, P = 0.179) (Figure 2B).

## Discussion

This study and the study conducted on primigravidae that preceded it [2] examined the effects of HIV infection on both total IgG to CS2_VSA_ and phagocytic antibodies to CS2_VSA_ using histopathology to define malaria in pregnancy. We were particularly interested in associations between antibody responses and clinical outcomes in secundigravidae, since important associations between antibody levels and anaemia or low birth weight have been reported in this group [10]. Limited sample numbers restricted our ability to further divide the women according to the different placental malaria stages, as was done with the previous study [2].

The mean haemoglobin level of participating women was low (mean = 10.6 g/dL), but the cause of this high prevalence of anemia is unknown, and likely to be multifactorial in this population [11]; haemoglobin levels did not differ between HIV-negative and HIV-positive women, nor between women with and without placental malaria. HIV infection, but not placental malaria, significantly influenced infant birth weight.

The correlation between total IgG to CS2_VSA_ and phagocytic antibodies was similar in strength to the correlation observed in responses of primigravidae from the same cohort [2]. Both phagocytic antibodies and total IgG to CS2_VSA_ were higher in PM+ women than in PM− women. HIV appeared to have significantly greater impact on phagocytic antibodies than on total IgG to CS2_VSA_ (Figure 1). This impact of HIV supports the hypothesis that, in cohorts of women infected with HIV, IgG sub-classes that contribute to phagocytosis are more impaired than those that do not, as we had suggested in our previous study [2]. Several studies have shown that different functional antibodies and even levels of total antibodies do not always correlate well with each other when controlling for gravidity or HIV [3], [6], [12]. Not all the sub-classes of IgG contribute to the process of phagocytosis. Several studies have demonstrated that the sub-classes IgG1 and IgG3 (called cytophilic classes due to their high capacity to induce immune cells to respond) are the most prominent classes among individuals living in malaria endemic areas that have achieved partial immunity [13] and are also the sub-classes that elicit phagocytosis in monocytes and macrophages [3], [14], [15]. It is worthwhile to point out that the remaining sub-classes of IgG, IgG2 and IgG4, have been associated with protection from malaria symptoms and with susceptibility to it, respectively [16], [17], [18]. It is thought that these antibodies, especially IgG4 might compete with the cytophilic antibodies for epitopes on the surface of the pRBC. In light of this, and because no analysis of the specific sub-classes of IgG and their possible functions (ADCI, anti-adhesion, anti-agglutination, anti-rosetting, phagocytosis, etc) was performed we cannot really reach a conclusion to what the function of the remaining IgG that are not phagocytosis contributors. Also, it should be noted that our findings may result from characteristics of the techniques involved to measure those antibodies, which in itself is important to point out. Not only is phagocytosis a more biologically relevant measure than total IgG, but it may be a more sensitive one also. When combined with our previous study, our data show that HIV affects phagocytic antibodies more than total IgG to CS2_VSA_ and that it does so to a greater extent in secundigravidae (this study) than in primigravidae.

When the women were divided according to presence or absence of PM, levels of total IgG to CS2_VSA_ were not associated with birth weight or haemoglobin levels in either group. On the other hand, phagocytic antibodies to CS2_VSA_ showed an association with birth weight, and within PM+ women this association remained after controlling for HIV, maternal age and maternal weight. Infant birth weight increased 3.57 g per unit of antibody measured (coef = 3.57, *p* = 0.025). Our observations have similarities to those of Duffy et al, who measured anti-adhesion antibodies and identified secundigravidae as a particular important group in the search for associations between antibodies and outcomes [10]. Being in a position of transition between susceptible primigravidae and immune multigravidae, secundigravidae present a very interesting focus of research. Although the numbers of women were not enough to completely dissect the role of these antibodies according to the different PM groups, the fact that in the ‘No malaria’ group HIV positive women had lower levels of phagocytic antibodies than did HIV negative women (with no significant differences for total IgG to CS2VSA) seems to confirm the results obtained in the PG group for women with past infection obtained in our previous study [2].

This study, although recognisably limited by the number of samples available, shed light into important areas of interest in the field of acquired immunity to malaria in pregnancy. First it revealed that phagocytic antibodies may be a more accurate measure of protective immunity than total IgG to CS2_VSA_. Second, it demonstrated that HIV has a bigger impact on phagocytic antibodies than on the total level of IgG to CS2_VSA_. Finally, it showed us that secundigravidae, who are acquiring protective immunity, can be an important group for the detection of associations between antibodies and clinical outcomes. These points are of particular interest when designing studies looking at the development of immunity to malaria in pregnancy in settings of HIV co-infection, where most vaccine trials are taking place.
